# Preparation, Characterization and Tailoring Properties of Poly(Vinyl Chloride) Composites with the Addition of Functional Halloysite–Lignin Hybrid Materials

**DOI:** 10.3390/ma15228102

**Published:** 2022-11-16

**Authors:** Jolanta Tomaszewska, Martina Wieczorek, Katarzyna Skórczewska, Izabela Klapiszewska, Krzysztof Lewandowski, Łukasz Klapiszewski

**Affiliations:** 1 Faculty of Chemical Technology and Engineering, Bydgoszcz University of Science and Technology, PL-85326 Bydgoszcz, Poland; 2 Faculty of Civil and Transport Engineering, Poznan University of Technology, PL-60965 Poznan, Poland; 3 Faculty of Chemical Technology, Poznan University of Technology, PL-60965 Poznan, Poland

**Keywords:** poly(vinyl chloride), halloysite–lignin hybrid fillers, structural and physicochemical characteristics, thermal and mechanical properties

## Abstract

In this article, halloysite–lignin hybrid materials (HL) were designed and obtained. The weak hydrogen bonds found between the components were determined based on Fourier transform infrared spectroscopy (FTIR), proving the achievement of class I hybrid systems. The HL systems were characterized by very good thermal stability and relatively good homogeneity, which increased as the proportion of the inorganic part increased. This was confirmed by analyzing scanning electron microscope (SEM) images and assessing particle size distributions and polydispersity indexes. Processing rigid poly(vinyl chloride) (PVC) with HL systems with a content of up to 10 wt% in a Brabender torque rheometer allowed us to obtain composites with a relatively homogeneous structure confirmed by SEM observations; simultaneously, a reduction in the fusion time was noted. An improvement in PVC thermal stability of approximately 40 °C for composites with HL with a ratio of 1:5 wt/wt was noted. Regardless of the concentration of the HL system, PVC composites exhibited inconsiderably higher Young’s modulus, but the incorporation of 2.5 wt% of fillers increased Charpy impact strength by 5–8 kJ/m^2^ and doubled elongation at break. This study demonstrated that favorable mechanical properties of PVC composites can be achieved, especially with an HL system with a ratio of 5:1 wt/wt.

## 1. Introduction

PVC has become one of the major commodity thermoplastics, with a global world market of approximately 38 million tonnes in 2020 [1]. Due to favorable properties, such as good mechanical strength, fire retardant properties, chemical and environmental resistance, widely developed processing capabilities, relatively low price, as well as susceptibility to modification of properties, PVC is widely used in various applications such as building products (including window frames and other profiles), piping (including water and sewerage pipes), packaging, insulation and sheathing for low voltage power supplies, automotive applications and medical products [2,3,4]. To modify the properties of PVC for specific applications, a wide assortment of additives are used, including heat and UV stabilizers, plasticizers, processing aids, impact modifiers, lubricants and fillers [4]. The latter include widely employed fillers of natural origin, both organic and inorganic, the employment of which allows for the production of environmentally friendly natural composites [5,6,7,8,9,10,11,12,13], whereby the influence of these fillers on the processing, mechanical and thermal properties of PVC depends on many factors, including their origin, form, particle size, concentration in the polymer matrix, as well as modification of filler surface and used coupling agents [11,12,13].

Applications of lignocellulosic materials, including wood fiber and wood flour [14,15,16,17,18,19,20], as well as agricultural residues [21,22] for the manufacture of PVC matrix composites, have also been described in the literature. Because of its renewable and biodegradable nature and favorable properties such as high availability, low weight, reinforcing capability, as well as antimicrobial and antioxidant, lignin is one of the materials with the highest application potential for the development of novel polymer composite materials [23,24] including those based on PVC [25,26,27]. Furthermore, lignin is the second most abundant natural polymer, preceded only by cellulose [23,24]. In addition to the use of lignin in the development of composite materials, other industrial applications of this biopolymer are very important, such as flame retardants, dispersants, surfactant formulations, seed coatings, wood adhesives, etc. [28,29,30,31].

According to Feldman and Banu [25], the intermolecular interactions between the polymer and lignin in rigid PVC blends can take place only at low lignin concentrations (up to 10 parts) due to the tendency of lignin to agglomerate at high loadings. Moreover, based on the results of mechanical tests and SEM observations of plasticized copolymer (vinyl chloride-vinyl acetate) filled with a hardwood organosol lignin, it was stated that specific plasticizers could dissociate part of the H-bonds present in lignin, thus creating possibilities of interaction with other polar polymers by intermolecular bonds. Liu et al. reported that the addition of lignin up to 30 phr into plastified copolymer by 20 phr of dioctyl phthalate PVC blend changes the rheological properties during kneading in a Haake mixer because the rigidity of lignin particles leads to more intensive shearing in the system. The mechanical properties of the blends were noted as being worse than that of neat PVC; however, these properties, especially toughness, were improved after lignin was treated with poly(ethyl acrylate-co-maleic anhydride) [26]. In related work, Yong et al. noted the possibility of using lignin to improve the hydrophilicity and antifouling properties of PVC membranes. Based on the results of SEM, XRD (X-ray diffraction) and DSC (differential scanning calorimetry) analyses, they concluded that PVC is compatible with lignin and homogeneous PVC/lignin sol can be formed to prepare membranes via the phase inversion method [27].

Although lignin-based hybrid materials are increasingly being used to modify polymers [32,33,34,35,36], the applications of such functional hybrid materials as fillers for the production of PVC composites are not widely reported in the available literature [37,38,39,40,41,42,43]. The influence of modified materials with a surfactant layered double hydroxide (LDH) and halloysite nanotube (HNT) on the thermal properties of PVC composites with reed flour was described by Dahmardeh Ghalehno et al. [37]. They found improvement in the thermal stability and an increase in the crystallization temperature and melting temperature of the composites through the addition of both types of nanoclays. In terms of improving combustion properties, i.e., increase in time to ignition and limiting oxygen index values, HNT was more effective compared to LDH.

The authors of the study [41,42,43] investigated the influence of lignin (L) combined with 3.5 µm silica (S), as well as eco-friendly magnesium hydroxide–lignin (Mg(OH)_2_) hybrid material on the properties of unplasticized PVC. It should be stated that it is possible to obtain a PVC composite containing up to 10 wt% of both types of hybrid fillers using a melt processing method with homogeneous dispersion of filler in the matrix. The presence of fillers contributes to the favorable processing and performance properties of the composites, in particular, to increase the Vicat softening temperature (VST) and to facilitate the improvement of the thermal stability. This is an important parameter due to the possibility of safely producing materials by conventional processing methods. Moreover, both PVC/S–L and PVC/Mg(OH)_2_-L composites exhibited a higher Young’s modulus than neat PVC, without a notable reduction in tensile strength. Additionally, the introduction of the Mg(OH)_2_-L system into the PVC matrix caused an increase in the oxygen index [43].

Due to the beneficial effect of lignin-based functional hybrid fillers on the processing, structural, thermal and mechanical properties of unplasticized PVC matrix composites that we demonstrated in our earlier works [41,42,43], and given the growing demand in the plastics market for eco-friendly materials based on available mineral and plant materials, further research into the development of a new hybrid filler as a filler for a large-volume polymer such as PVC is warranted. The use of halloysite as the second component of this filler in the present study is due to its high availability and unique structure and properties, which makes it a widely used filler for polymeric plastics, including PVC, positively affecting their thermal and mechanical properties [44,45,46,47,48,49,50,51,52,53,54,55].

The literature also describes the use of halloysite for the production of biocomposite films with cellulose fibers [56] and as a component of hybrid fillers in a system with cellulose used to improve the properties of epoxy nanocomposite coatings [57,58], vinyl-ester composites [59] and phenolic foams with beneficial thermal insulation and flame retardancy [60]. Krishnaiah et al. showed that chemical surface modification of both sisal fibers and halloysite nanotubes leads to higher values of tensile strength and modulus by 55% and 50%, respectively, as well as higher dynamic moduli compared to that of pure PP. Additionally, an improvement in thermal stability by 60 °C for the composites containing surface-treated hybrid fillers was found [61].

Previous research and the conclusions drawn from them, as well as the analysis of the available literature, prompted the authors of this work to design and obtain innovative halloysite–lignin hybrid materials and then use them as poly(vinyl chloride) fillers. The authors believe that there are no such systems in the current literature on the subject. The obtained products were subjected to a wide range of measuring activities, ranging from determining the most important physicochemical and dispersion-microstructural properties of fillers to the evaluation of polymer composites (processing properties, evaluation of thermal stability, mechanical properties and structural evaluation).

## 2. Materials and Methods

### 2.1. Materials

Halloysite nanoclay (Al_2_Si_2_O_5_(OH)_4_·2H_2_O), with a molecular weight equal to 294.19 g/mol and the CAS number: 1332-58-7, was purchased from Merck, Darmstadt, Germany, and kraft lignin purchased from Merck, Darmstadt, Germany (average M_w_ ~10,000 g/mol, CAS number: 8068-05-1) were used in these studies as fillers.

The rigid PVC dry blend is composed of a suspension of PVC S-61 Neralit (Spolana Anwil Group, Neratovice, Czech Republic) 100 phr and organotin stabilizer Patstab 2310 (Patcham, Goor, The Netherlands) 4 phr, and 1 phr Naftolube FTP paraffin wax (Chemson, Arnoldstein, Austria) was employed as the matrix for the composites. Stabilizer and paraffin wax were the only added components in order to minimize the influence of additives on processing properties.

### 2.2. Preparation of Halloysite–Lignin Materials

The halloysite–lignin materials were obtained using a mechanical method. This method is environmentally friendly and does not require harmful and dangerous additional compounds.

In the first stage, a given amount of the inorganic component, halloysite, was placed in an RM100 mortar grinder (Retsch GmbH, Haan, Germany) with an appropriate amount of lignin. The process of grinding the ingredients with simultaneous mixing was carried out for 1 h. The system was then transferred to a high-energy planetary ball mill (Pulverisette 6 Classic Line, Fritsch GmbH, Amberg, Germany) for intensive milling and mechanical alloying of the powder materials. The use of both devices served to achieve the best possible homogeneity of the final product and to obtain a class I hybrid system by creating appropriate interactions, mainly of a physical nature, between the component. The final products in the form of halloysite–lignin materials were produced at three different ratios: (i) 1 part by weight of halloysite per 5 parts by weight of lignin (H1L5); (ii) 1 part by weight of halloysite per 1 part by weight of lignin (H1L1) and (iii) 5 parts by weight halloysite per 1 part by weight of lignin (H5L1).

### 2.3. Characteristics of Halloysite–Lignin Materials

The morphology and microstructure of the powder materials were analyzed using photomicrographs obtained with a Tescan VEGA3 scanning electron microscope (SEM) (Tescan Orsay Holding a.s., Brno, Czech Republic).

Particle size distribution analysis was examined by utilizing the Zetasizer Nano ZS apparatus (Malvern Instruments Ltd., Malvern, UK) according to the non-invasive back scattering method. This method measures particles from 0.6 to 6000 nm. As part of our work, the particle size distribution was assessed in isopropanol as a dissolution medium.

To determine the presence of functional groups in the structure of pristine components (halloysite and lignin) and in the obtained hybrid materials, samples were subjected to Fourier transform infrared spectroscopy. FTIR spectra were obtained using a Vertex 70 spectrometer (Bruker Optics GmbH & Co. KG, Ettlingen, Germany). Materials were analyzed in the form of KBr tablets, prepared by mixing 250 mg of anhydrous KBr and 1 mg of the analyzed substance under a pressure of 10 MPa. The FTIR analysis was performed at a resolution of 0.5 cm^−1^ in a wavenumber range of 4000–450 cm^−1^ (number of scans: 64).

A Jupiter STA449 F3 apparatus (Netzsch, Selb, Germany) was used to perform thermogravimetric analysis (TGA). In the tests, 10 mg samples were placed in an Al_2_O_3_ crucible, then heated in a nitrogen atmosphere at a rate of 10 °C/min, from 30 °C to 1000 °C, with a gas flow rate of 40 cm^3^/min.

A Zetasizer Nano ZS apparatus (Malvern Instruments Ltd., Malvern, UK) was employed to measure the zeta potential, which, through measurement of electrophoretic mobility via laser Doppler velocity (LDV), enables the calculation of the zeta potential using the Henry equation. The measurement took place in the pH range from 2 to 10, in the presence of 0.001 M NaCl electrolyte, which allowed the determination of the electrokinetic curves. The measurement was carried out three times, and the final value was the average value. The mean standard error of the zeta potential measurement was ±2 mV, while the pH measurement error was ±0.1.

### 2.4. Preparation of PVC/Halloysite–Lignin Polymer Composites

Poly(vinyl chloride) composites with hybrid fillers and raw halloysite and lignin were obtained by mixing in a molten state. In the first stage, PVC compounds with fillers previously dried for 3 h at the temperature of 105 °C were prepared by mixing with a high-shear mixer. Afterwards, the compounds were processed by kneading using the FDO 234H type Brabender plastographometer (Brabender GmbH & Co. KG, Duisburg, Germany) with a temperature of chamber wall of 190 °C; a rotational blade speed of 30 rpm and friction of 1:1.5 were also employed. The charge weight was 60 g in each case. Kneading was performed for 10 min. The unfilled PVC dry blend was processed under similar conditions as the reference sample.

The processed materials were ground in a milling grinder of the authors’ own design, and the resulting ground material was pressed at the temperature of 190 °C under a maximum working pressure of 150 bar. The obtained 2 and 4 mm thick molded pieces were used to cut out specimens for mechanical testing (tensile test, impact strength and hardness) and thermal measurements (Vicat softening temperature) using a Seron 6090 numerical milling plotter (Seron, Stalowa Wola, Poland). The compositions of the PVC mixtures and their abbreviations are presented in Table 1.

### 2.5. Characteristics of PVC/Halloysite–Lignin Composites

#### 2.5.1. Processing Properties

During the kneading of the compounds, the changes in the torque of the rotors as a function of time were recorded. Based on the obtained plastograms, values characterizing the processing properties were determined, namely: the maximum value of the torque (M_X_), the time of its attainment (t_X_), as well as torque at the end stage of kneading (M_E_).

#### 2.5.2. Thermal Properties

The thermal stability of PVC composites was determined by the Congo red test and thermogravimetric analysis (TGA). The thermal stability test using the Congo red method was carried out according to the ISO 182-1:1990 standard [62] at 200 °C. The TGA measurements were performed using an analyzer TG 209 F3 (Netzsch, Selb, Germany) in an open ceramic crucible, operating at a scanning rate of 10 °C/min, in the temperature range from 30 °C to 700 °C under a nitrogen-protective atmosphere. For every composition, three TGA measurement procedures were repeated. The temperature of 1%, 5%, 10% and 50% weight loss, labeled, respectively, as T_1%_, T_5%_, T_10%_ and T_50%_, as well as residual mass after decomposition at 700 °C (RM), were analyzed. The Vicat softening temperature tests were carried out on samples with dimensions of 10 mm × 10 mm × 4 mm according to the ISO 306: 2006 standard [63] by employing an RV-300A HDT&Vicat Tester (Jinan, Shandong, China). For every sample type, the measurements were carried out in triplicate.

#### 2.5.3. Mechanical Properties

The static tensile test was performed with a Zwick Roell Z010 universal testing machine (Zwick GmbH & Co. KG, Ulm, Germany) according to ISO 527-1, using type 1BA samples [64]. The test was carried out at room temperature and at a tensile rate of 1 mm/min in the range of modulus of elasticity determination and then of 5 mm/min. Modulus of elasticity (E_t_), stress at yield (σ_Y_), tensile strength (σ_M_) and strain at break (ε_B_) were determined. The measurements were carried out for 10 samples of each material.

The Charpy impact strength without notch was tested by means of a HIT5P device from Zwick Roell (Zwick GmbH & Co. KG, Ulm, Germany), according to the PN-ISO 179-1 standard [65].

Dynamic mechanical properties were analyzed through an Artemis DMA 242 D Netzsch apparatus in torsion mode, operating at a frequency of 1 Hz, at a heating rate of 2 °C/min within the range of temperature between 25 °C and 100 °C. Storage modulus (E′) and loss factor (tanδ) were determined. The temperature by which the loss factor reveals the maximum value was taken as the glass transition temperature.

#### 2.5.4. Microscope Observations

To determine the composite structure, observations of PVC composites with 1 wt% of filler were carried out by means of scanning electron microscopy using a Tescan VEGA3 (Tescan Orsay Holding a.s., Brno, Czech Republic).

## 3. Results and Discussion

### 3.1. Characteristics of Halloysite–Lignin Materials

#### 3.1.1. Particle Size Distribution and Microscope Observations

**Figure 1:**
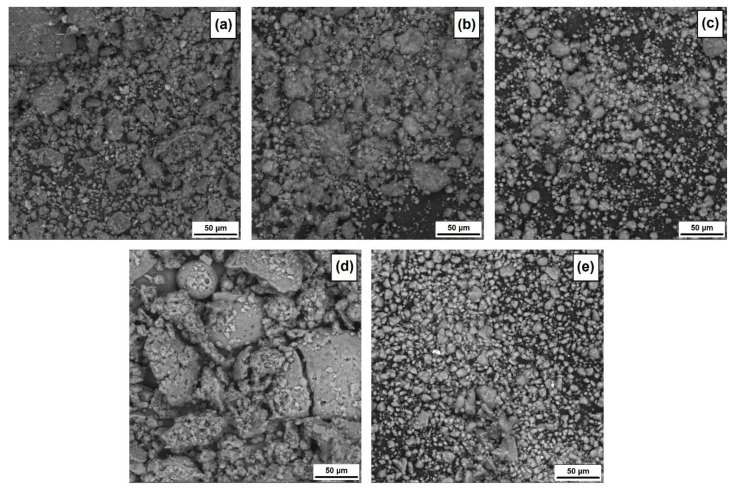
SEM images of halloysite–lignin hybrid materials: 1:5 wt/wt (**a**), 1:1 wt/wt (**b**) and 5:1 wt/wt (**c**) and pristine components lignin (**d**) and halloysite (**e**).

As part of this article, the microstructural, dispersion and physicochemical properties were assessed. Figure 1 presents SEM images of the produced halloysite–lignin hybrid systems and pristine components (lignin and halloysite). In addition, Table 2 shows the particle size distributions for the analyzed products and the corresponding polydispersity indexes (PdI).

Based on the SEM images (see Figure 1), it can be concluded that the obtained hybrid systems are characterized by relatively good homogeneity. In individual hybrids, one can see the presence of single primary particles of nanometric size (which was additionally confirmed by examining the particle size distributions, see Table 2), which tend to form aggregates and, consequently, agglomerates. This tendency is visible as the ratio of the relevant components increases, which can be clearly seen based on the SEM images and the results presented in Table 2.

The widest particle size distribution (164–342 nm and 459–6439 nm), and thus the lowest homogeneity (PdI = 0.988), is characteristic of a pristine lignin sample. Additionally, lignin, as a result of intensive grinding in a ball mill, created secondary agglomerates that are clearly visible in the SEM microphotography (see Figure 1d). Halloysite, although much more homogeneous, also tends to form larger particle clusters (see Figure 1e and Table 1).

Earlier research conducted by our research group on other hybrid materials has already confirmed the conclusions and tendencies mentioned above [32,33,34,35,36].

#### 3.1.2. FTIR Spectroscopy

**Figure 2:**
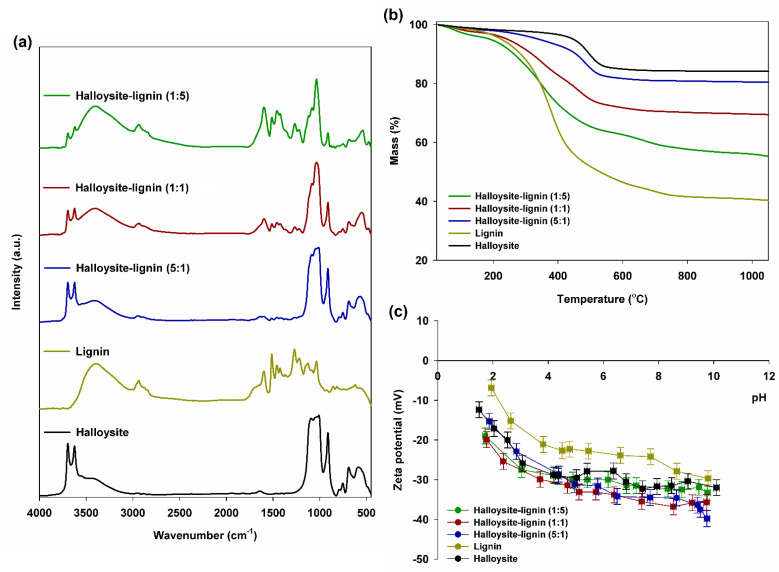
FTIR spectra (**a**), TGA curves (**b**) and zeta potential versus pH (**c**) of halloysite–lignin hybrid materials and pristine components.

Based on the analysis of FTIR spectra, characteristic functional groups for the components (lignin and halloysite) and the obtained hybrid materials were identified. Figure 2a presents FTIR spectra for lignin, halloysite and halloysite–lignin systems with different ratios of individual components.

The spectrum of halloysite is characterized by vibration ranges from its octahedral and tetrahedral layers. Based on the analysis of the obtained spectrum, it can be concluded that at the maximum wavenumbers of 3695 cm^−1^ and 3622 cm^−1^, vibrations of the hydroxyl groups present on the surface of the octahedral layer occur. Vibrations in the range of ~1300–1100 cm^−1^ are vibrations of the tetrahedral aluminosilicate layer. In turn, the band originating from the bending vibrations of Si-O-Si bonds in the plane occurs in the range of 1080–1000 cm^−1^. Moreover, the maximums at 910 cm^−1^ and 560 cm^−1^ correspond to the tensile vibrations of Si-O-Al bonds, and the maximums of 750 cm^−1^ and 680 cm^−1^ do so to the vibrations of Si-O bonds [51,66,67].

Figure 2a also shows the spectrum of pristine lignin. The spectrum shows the presence of the following bands: O–H stretching vibrations bands (phenolic –OH + aliphatic –OH) in the range of 3600–3200 cm^−1^ and C–H stretching vibrations in the range of 2960–2920 cm^−1^ (CH_3_ + CH_2_) and 2850–2840 cm^−1^ (OCH_3_). The wider band in the range of 1710–1550 cm^−1^ results from the overlapping of consecutively unbound and bound bonds of stretching vibrations of the carbonyl group C=O at wavenumber 1610 cm^−1^ and successively 1508 cm^−1^ and 1458 cm^−1^, and of stretching vibrations of C-C bonds of the aromatic skeleton. In the case of the lignin FTIR spectrum analysis, the bands with the maximum intensity for the wavenumbers, respectively, 1370 cm^−1^, 1255 cm^−1^ and 1225 cm^−1^, corresponding to the stretching vibrations of the C–O, C–O(H) + C–O(Ar) bonds, are also important. The presence of C–O–C ether bonds is indicated by the band of stretching vibrations at the wavenumber of 1045 cm^−1^. The last group of significantly characteristic lignin bands are bands in the plane of deformation Ar C–H (1140 cm^−1^) and outside the plane Ar C–H (bands with a wavenumber below 1000 cm^−1^, including 852 cm^−1^, 814 cm^−1^ and 790 cm^−1^). All data relating to the FTIR analysis of pristine lignin is consistent with the available literature [32,33,34,35,36,42,43].

In the case of the spectra of hybrid fillers (see Figure 2a), maxima characteristics for pristine components, i.e., lignin and halloysite, can be observed. Additionally, slight shifts of the maxima of individual bands of characteristic groups can be noticed, related to the weak physical interactions between the components. This allows us to conclude the creation of class I hybrid material. We came to similar conclusions when analyzing other hybrid materials described in previous articles [32,33,34,35,36,42,43].

#### 3.1.3. TGA Analysis

As part of the characteristics of the manufactured products, thermogravimetric curves of hybrid fillers and pristine components were determined (see Figure 2b).

The TGA curve for halloysite shows a gradual decrease observed at 30–400 °C. This is related to the dehydration process and the gradual loss of water between the layers of the halloysite. Another strong decrease visible in the temperature range 400–600 °C indicates dehydroxylation of AlOH, which is consistent with the dehydroxylation temperature of halloysite, occurring at approximately 450–600 °C.

Figure 2b also shows the thermogravimetric curve determined for lignin. The first stage, associated with a slight 10% weight loss (in the temperature range of 25–210 °C), is mainly related to the local elimination of water bound on the lignin surface. The second step of high weight loss of approximately 35% in the temperature range of 200–600 °C is related to the complicated thermal decomposition of the compound, including the formation of new bonds as a result of the cross-linking reactions taking place. Thermal treatment above 600 °C (up to 1000 °C) in the third stage is associated with molecule fragmentation as a result of unclear and uncontrolled reactions. These data are in line with those of previously published articles [32,33,34,35,36,42].

When assessing the thermal stability of halloysite–lignin hybrid fillers, it can be concluded that the thermal stability of the final product decreases with increasing biopolymer content. In the initial temperature range (processing range), the changes are not that visible, but above approximately 200 °C, these changes are very significant. For the halloysite–lignin hybrid material (1:5 wt/wt), the weight loss is 41%, for a product with a component ratio of 1:1 wt/wt, this loss is 30%, while for the hybrid system with 5:1 wt/wt, this loss is 19%.

The favorable results obtained at this stage of the research demonstrate that halloysite–lignin hybrid materials can be successfully used as a new generation of environmentally friendly and relatively cheap polymer fillers.

#### 3.1.4. Zeta Potential Analysis

In order to determine the electrokinetic stability of the products used, the zeta potential of the tested fillers was assessed. Figure 2c shows the dependence of the zeta potential on the pH value in the range of 2–10. We found that halloysite and halloysite–lignin hybrid materials show good electrokinetic stability for pH > 3, while lignin reveals this for pH > 4. Additionally, as can be seen based on the obtained test results presented in Figure 2c, a decrease in the electrokinetic potential for all materials was observed with the increase in the alkaline character.

Based on the conducted research, it can therefore be unequivocally concluded that the share of halloysite in the hybrid material improves the electrokinetic stability of the designed and manufactured hybrid fillers. We obtained analogous results in our previous work by testing hybrid fillers combining silica with lignin [35,36,68] or zinc oxide with lignin [33,34].

### 3.2. Characteristics of PVC/Halloysite–Lignin Composites

#### 3.2.1. Processing Properties

During the processing of PVC in a molten state, PVC grains transform into the compact form of the final product through heat, pressure and shearing. This transformation is called ‘PVC gelation’ and consists of the gradual disintegration of PVC grains, their plasticization and fusion. To characterize the progress in PVC gelation, torque measurements during kneading in a Brabender rheometer chamber were undertaken and recorded in the form of plastograms [22,69].

**Figure 3:**
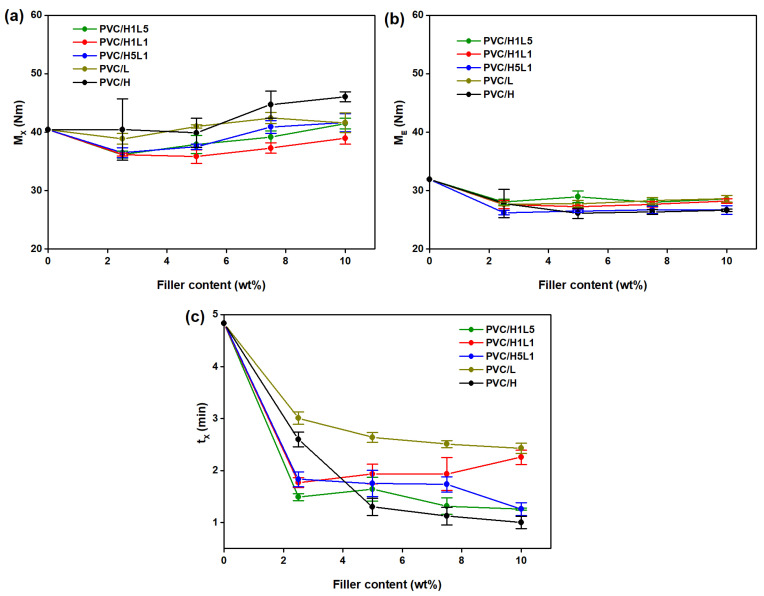
The fusion properties of the PVC composites: (**a**) maximum value of torque (M_X_), (**b**) values of torque at the end point of plastograms (M_E_) and (**c**) time required to reach the M_X_ (t_X_), as a function of filler content.

**Figure 4:**
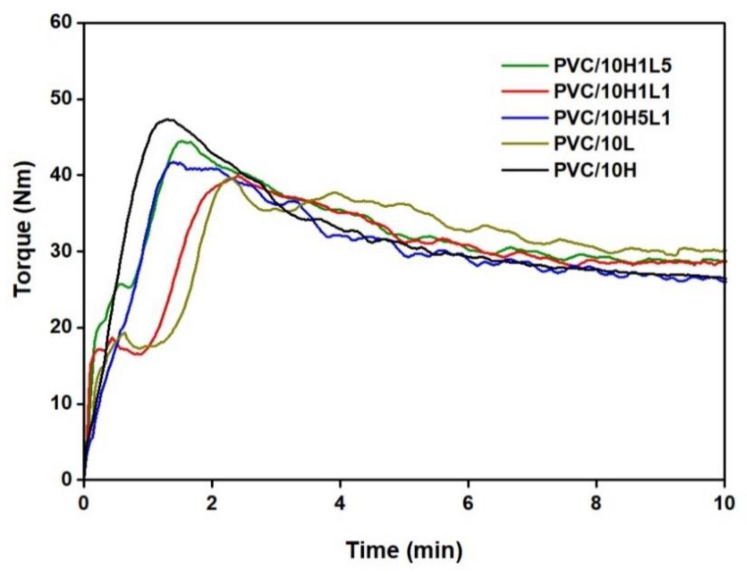
Torque vs. time of kneading for PVC and PVC composites with 10 wt% of fillers: lignin, halloysite and halloysite–lignin hybrids.

The study analyzed the characteristic values of torque read out from plastograms. We measured maximum torque at the gelation point (M_X_), also called ‘fusion’ or ‘gelation torque’, torque at the end stage of kneading (M_E_), as well as time to reach maximum torque (t_X_) so-called ‘gelation’ or ‘fusion time’ [38] (see Figure 3a–c). The course of torque dependence on kneading time of PVC and PVC composites with 10 wt% content of all types of fillers, shown in Figure 4, is characteristic of the processing of unplasticized PVC. The occurrence of the maximum value of torque is related to the gelation effect and is indicative of correctly selected processing conditions [22,69].

The value of the maximum torque of kneaded mixtures of PVC with hybrid fillers, regardless of the proportions of the two components of the hybrid filler, i.e., lignin and halloysite, and with lignin alone, is similar to, or lower by a maximum of approximately 5 Nm, compared to the M_X_ value of unfilled PVC of 40 Nm (see Figure 3a). The effect of lowering the M_X_ value compared to PVC is particularly evident when the content of the L and HL fillers is 2.5 wt%; a further increase in their concentration up to 10 wt% results in a slight increase in Mx, with practically all cases not exceeding the M_X_ value of unfilled PVC. A similar trend was observed for PVC blends containing halloysite, but the introduction of 7.5 wt% and 10 wt% of this filler resulted in approximately 3 and 6 Nm higher torque, respectively, compared to unmodified PVC.

Analysis of the data in Figure 3b shows that the introduction of 2.5 wt% fillers into the PVC matrix, regardless of the type, results in a reduction in M_E_ values of approximately 3.5–5.5 Nm compared to unfilled PVC, for which the value is 32 Nm. An increase in the concentration of fillers up to 10 wt% does not change the torque at the end stage of kneading. It can be concluded that the fillers used, introduced in amounts up to 10 wt%, do not strongly affect the viscosity of the mixtures in the viscous state.

The introduction of fillers into the polymer matrix, regardless of the type, results in a reduction in the time required to reach the maximum torque value (see Figure 3c). Mixtures with hybrid fillers with the lowest concentration used in the study, i.e., 2.5 wt%, show a shorter fusion time by approximately 3 min compared to unfilled PVC; the same content of halloysite reduces t_X_ by 2.2 min and lignin by 1.5 min. With an increase in the concentration of halloysite to 10 wt%, the gelation time decreases sharply to 1 min and is the shortest compared to all other blends; it is also almost five times shorter compared to neat PVC. In the case of lignin, there is a slight tendency to shorten the time with increasing concentration and the t_X_ value for PVC/10L is two times lower than that of unfilled PVC.

In investigating the effect of SiO_2_ particle size on the fusion behavior of PVC composites, Ari and Aydin [70] found that the increase in fusion torque and reduction in fusion time is associated with a significantly higher number of SiO_2_ nanoparticles in the same mixture volume compared to PVC microparticles, resulting in an increase in frictional forces. Based on the explanations for the fusion behavior of PVC mixtures with SiO_2_ with different particle sizes, as proposed by Ari and Aydin [70], it can be assumed that the significantly different torque and time gelation values found in our experiment for PVC/H and PVC/L mixtures are related to the different particle sizes of halloysite and lignin. At the same filler content, the number of nano-sized halloysite particles in contact with the hot surface of the chamber walls was much higher than that of large particles of lignin. Because of the large number and surface area of the heated nanoparticles, heat transfer to PVC filled with halloysite was faster than that to PVC filled with lignin. Consequently, quicker gelation occurred in the PVC/H composites, and gelation time was shorter. The increase in the friction in the system and the increase in the transfer of heat and shear throughout the PVC grains was also described by Chen et al. for PVC composites with carbon black [71]. These effects were responsible for the increase in the fusion torque of the PVC/carbon black composite and the consequent reduction in time with the increase in carbon black filler concentration in the system.

We observed similar effects during processing studies of PVC blends with silica and lignin, while the addition of silica or silica–lignin hybrid filler caused a substantial increase in torque compared to halloysite and halloysite–lignin [41]. The favorable shortening of the gelation time was also found in our processing studies of PVC composites with the addition of the Mg(OH)_2_–lignin hybrid filler; however, no significant increase in the mechanical and thermal loads of the processed compounds was observed [43].

#### 3.2.2. Thermal Properties

The thermal stability of PVC and PVC matrix composites was evaluated based on the results of thermogravimetric analysis and the Congo red test. Its determination is very important for the safe processing of PVC in the molten state due to the possible instability of the polymer under temperature and shear conditions leading to dehydrochlorination reactions and progressive degradation [72].

**Figure 5:**
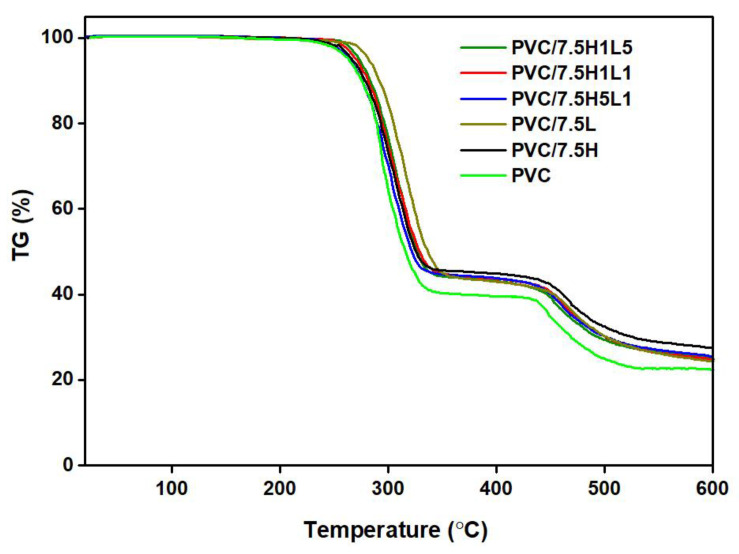
Exemplary TGA thermograms of PVC and PVC composites with 7.5wt% of fillers: lignin, halloysite and halloysite–lignin hybrids.

The TGA thermograms of PVC and composites with all types of fillers are essentially similar and show two major degradation stages in the temperature ranges 270–350 °C and 420–550 °C, which are related to the decomposition of the polymer matrix (see Figure 5).

In the first degradation stage, the dehydrochlorination of PVC macromolecules takes place [73], and the evolved hydrogen chloride gas accelerates the progress of decomposition, leading to the formation of conjugated polyene sequences. The second decomposition stage lies within the temperature range 420–550 °C, where the chain scission of the carbonaceous backbone and cross-linking between chains occur, leading to the formation of a carbonized residual char [74].

In predicting the applicability of the developed materials, it is necessary to consider the influence of the proposed fillers on the thermal stability of composites under processing conditions, i.e., up to a temperature of approximately 200 °C [75]. Based on thermogram analysis, it was found that all the investigated materials were thermally stable in the processing range, and the first weight losses of the sample (T_1%_) were observed in the range of approximately 242–261 °C (see Table 3). In the case of PVC materials, it is important to evaluate the first destructive changes associated with the release of hydrogen chloride, which acts as a catalyst and generates a series of chain reactions leading to the matrix decomposition process [76]. Therefore, it is important to identify the first destructive changes in PVC as a matrix for composites (see Table 3).

Analysis of the 1%, 5%, 10% and 50% weight loss temperature values for PVC/L composites allowed us to conclude that lignin improves the thermal stability of PVC (see Table 3). The highest weight loss temperature values were obtained using the highest concentration of lignin in the polymer matrix. The difference between T_1%_, T_5%_ and T_10%_ values for PVC/L composites and unmodified PVC was approximately 10 °C, and in the case of T_50%_, approximately 14 °C.

The weight loss temperatures of PVC/H1L1 and PVC/H1L5 composites had similar values when comparing samples of the same concentration; the composites containing 10 wt% filler had the highest stability in both cases. The use of H1L1 and H1L5 hybrid fillers also had a favorable effect on the thermal stability of PVC; however, the increase in T_1%_, T_5%_, T_10%_ and T_50%_ compared to pristine PVC was not as large as in the case of PVC/L, as it ranged from 1.4 °C to 14.1 °C, depending on the filler concentration.

The composites modified with halloysite and hybrid filler with the highest proportion of this component showed lower stability compared to the other systems. The weight loss temperatures of PVC/H and PVC/H5L1 samples had similar values comparing samples with identical concentrations. The temperature of onset of decomposition taken as the T_1%_ of both types of composites was lower than that of unmodified PVC, regardless of the filler concentration. The T_5%_ and T_10%_ values were slightly higher (by approximately 3–4 °C) compared to PVC only at 2.5 wt% filler content.

Moreover, the thermal stability of these composites decreased as the amount of the filler in the polymer matrix increased. The reason for this behavior may be the different thermal conductivity of the mineral filler, which accelerated heat transfer to the PVC macromolecules and consequently resulted in lower thermal stability. Another explanation for this phenomenon may be the influence of the mineral filler on the processing effects of PVC blends which may also result in a deterioration of the thermal stability of the composites. Of note, a slight decrease in T_5%_ and T_50%_ values caused by the loading of the mineral filler in the form of nanoclays in the concentration of 6 phr in rigid PVC foams was determined by Moghri et al. [77].

Summarizing the presented results of the TGA analysis, it can be concluded that the use of halloysite–lignin hybrid fillers guarantees safe melt processing of composites without the risk of PVC degradation. Lignin, as a component of the hybrid system, compensates for the negative effect of halloysite on the thermal stability of PVC by showing a protective effect on the polymer when its content in the system is greater or the same as for the mineral filler. Thus, the negative impact of the mineral component on thermal stability visible in the initial step of decomposition, which is important from the point of view of safe PVC processing, can be reduced by its combination/hybridization with lignin. A similar protective effect against accelerated decomposition in the presence of inorganic fillers, i.e., SiO_2_ and Mg(OH)_2_, was described in our earlier works [41,42,43]. The reduction in HCl release during the pyrolysis of PVC with the biomass materials due to the presence of lignin as a component of biomass was reported by Zhou et al. It was concluded that biomass component materials and/or bio-char could act as catalysts that inhibit the dehydrochlorination process or promote the chain scission of PVC [78].

**Figure 6:**
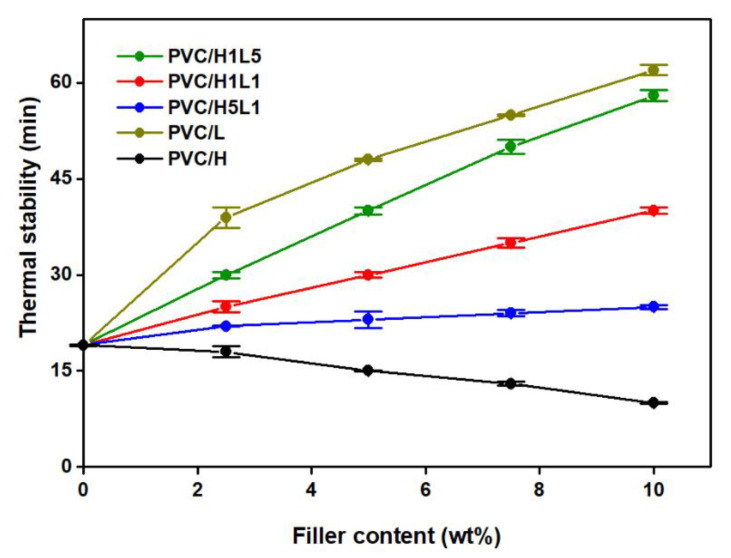
Thermal stability of PVC and PVC-based composites vs. filler content: lignin, halloysite and halloysite–lignin hybrid materials.

Figure 6 shows the thermal stability time determined by the Congo red method of PVC and PVC-based composites as a function of the filler content. Composites of PVC with lignin and with hybrid filler H1L5 and H1L1 had a much longer time of thermal stability compared to unmodified PVC, and the higher the lignin content in the hybrid filler, the longer this time. The increase in the thermal stability time was the highest when lignin alone was used; introducing only 2.5 wt% of lignin into the PVC matrix resulted in more than a two-fold extension of this time. Increasing the concentration of lignin and H1L5 and H1L1 hybrid fillers to 10 wt% resulted in a systematic extension of the thermal stability time to approximately 62 min in the case of PVC/L composite and, respectively, up to 58 min and 40 min in the case of PVC/H1L5 and PVC/H1L1 composites. A similar effect was found in our earlier works [41,42,43], where the presence of the lignin component in hybrid materials, both with silica and with Mg(OH)_2_, had a significantly beneficial effect on the results determined by the Congo red test thermal stability of composites based on unplasticized PVC matrix. The introduction of the Mg(OH)_2_/lignin filler to the PVC matrix in the amounts of 7.5 wt% and 10 wt% led to higher temperature stability compared to unfilled PVC by 29 min and 41 min, respectively [43]. With the same filler content, PVC/SiO_2_–lignin composites were also characterized by more than twice as high thermal stability compared to unfilled PVC [41]. In both cases, the improvement in thermal stability was greatest when the filler was native lignin; the time of thermal stability was then three times longer compared to neat PVC.

This effect was explained by the reaction between methoxy groups in phenolic rings in lignin and the hydrogen chloride released from PVC, which leads to chloromethane formation, as proposed by Czegeny et al. [79]. The increase in PVC thermal stability related to the introduction of lignin into the matrix as a component of the hybrid filler was also reported by Zhang et al. [39]. Here, the static thermal stability of PVC with mechanically activated lignin was 85.7% higher than that of pure PVC, probably due to the improvement in dispersion and the compatibility of lignin rigid particles with PVC that is related to their mechanical activation. Additionally, the complex consisting of mechanically activated lignin and magnesium borate hydrate showed a synergistic effect of improvement in thermal stability, as the PVC composite exhibited a thermal stability time 466% higher than that of pure PVC, indicating that this novel additive effectively inhibited the generation of HCl from the thermal decomposition of PVC. Zhao et al. [80] confirmed that the addition of lignin improved the thermal stability of PVC but noted that, simultaneously, this led to substantial deterioration of the mechanical properties. The solution to this problem was the modification of lignin by the esterification method, which made it possible to obtain PVC composites with satisfactory mechanical and thermal properties.

The incorporation of a hybrid filler to the PVC matrix with a halloysite to lignin ratio of 5:1 makes the time of thermal stability of composites and pure PVC practically comparable, regardless of the H5L1 filler concentration used. When raw halloysite with a concentration of 2.5 wt% was used, a slight deterioration in the time of PVC thermal stability was observed. However, we found that an increase in the halloysite content results in further deterioration of the thermal stability to 10 min (by 44%) for 10 wt% filler content in the matrix. The effect of reducing the thermal stability of composites due to the introduction of halloysite from the Dunino mine was seen in our earlier work [81] and explained by the presence of impurities, among others, iron oxides (iron (II) and iron (III)).

The introduction of lignin as a component of the hybrid filler is an effective approach to eliminating the problem of the negative influence of the mineral filler on the thermal stability of PVC and allows us to increase the application potential of PVC/halloysite–lignin composites. The time of thermal stability found in our research, which in the case of composites containing 10 wt% of each type of hybrid filler, exceeds 20 min and is sufficient for the safe processing of PVC composites in the molten state without the risk of polymer degradation.

It should be added that the thermal stability of the produced composites depends significantly on the composition of the basic PVC blend. In PVC processing, a broad assortment of thermal stabilizers are applied to avoid the degradation of the polymer, and the selection of an appropriate one to meet the user-specified criteria is quite a complex task and very important in the case of PVC composite production.

**Figure 7:**
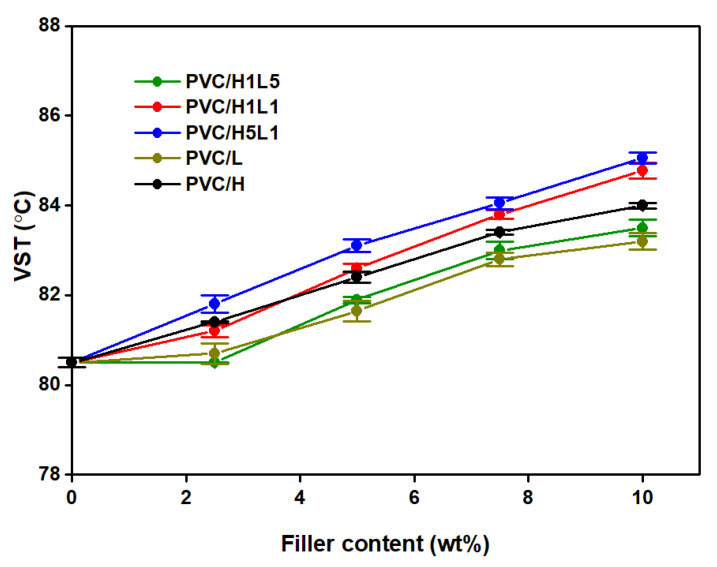
The Vicat softening temperature of PVC and PVC-based composites vs. filler content.

Figure 7 shows the VST of PVC composites as a function of filler content. The incorporation of fillers into the PVC matrix, regardless of the type, causes a slight increase in the Vicat softening temperature of the composites compared to pure PVC. The higher the value of this temperature, the higher the filler concentration.

Comparing the influence of individual hybrid filler components on softening temperature, halloysite is more effective in this regard than lignin; a 10 wt% concentration of this mineral in the matrix results in a 3.5 °C increase in VST values, while the same content of lignin results in a 2.7 °C increase. The highest softening temperature values are seen in composites containing filler with a halloysite–lignin ratio such as 5:1. Here, the increase in the VST value of the PVC/10 H5L1 composite compared to the unmodified matrix is 4.5 °C.

A similarly favorable softening temperature value was recorded for PVC/H1L1 composites, especially when the filler concentration was more than 5 wt%. Based on the analysis of the results shown in Figure 7, it can be concluded that the introduction of a hybrid filler into the PVC matrix with both equal proportions of halloysite and lignin and a dominant proportion of halloysite relative to lignin leads to a synergistic effect of improving the softening temperature.

The obtained effect of increasing the softening point, allowing for a potential extension of the scope of application of composites at increased temperature, especially in construction products, was also found in our earlier work [41]. The observed effect of the visible increase in the VST value was related to the incorporation of 7.5 wt% of silica and silica–lignin filler into the PVC matrix; VST values were higher in comparison with the origin PVC sample by as much as 10 °C in the case of PVC/silica–lignin and 14 °C for the PVC/silica composites.

#### 3.2.3. Mechanical Properties

**Figure 8:**
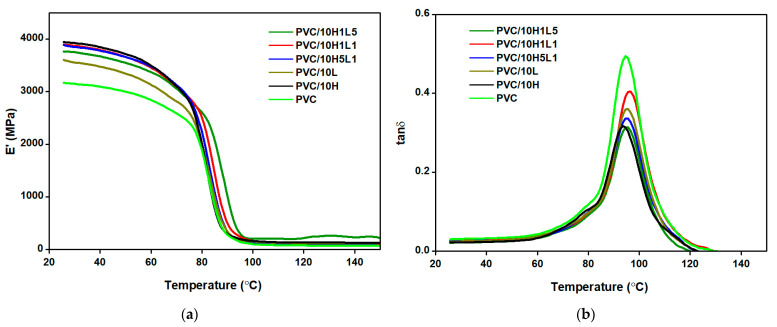
DMA thermograms of PVC and PVC composites with 10 wt% of fillers: storage modulus (**a**) and loss factor (**b**).

Figure 8a,b show the DMA thermograms obtained for PVC and composites containing 10 wt% of the fillers of all kinds. The values of the storage modulus (E’) determined at temperatures of 30 °C, 50 °C and 70 °C, as well as the glass transition temperature (T_g_) values, are summarized for all tested materials in Table 4.

The introduction of fillers in the form of halloysite, lignin and a halloysite–lignin hybrid filler caused an increase in the values of the storage modulus compared to PVC. Herein, the E’ values increasd as the filler concentration increased to 10 wt% in the matrix. The highest E’ values were recorded for composites with halloysite, indicating that the introduction of this filler into the matrix caused the greatest stiffness effect. The modulus values determined for PVC/10H are 4027 MPa, 3828 MPa and 3234 MPa, respectively, and are as much as 27–32% higher compared to the E’ values for PVC in the range up to 70 °C. The composites with lignin and hybrid fillers, regardless of the proportions of the two components, showed storage modulus values lower than PVC/H, with a different relationship of E’ as a function of concentration. In the case of PVC/L composites, the storage modulus initially increased to 3514 MPa as the lignin concentration increased to 5 wt% and practically did not change with further filler increased to 7.5 wt% and 10 wt%. The E’ moduli of PVC/H1L1 and PVC/H1L5 composites containing 2.5 wt% and 5 wt% filler were in the range of approximately 3190 MPa to 3250 MPa, after which their value increased by approximately 500 MPa for composites with 7.5 wt% and 10 wt% loading of filler, falling in the range of 3702–3749 MPa. An increase in the concentration of halloysite and filler with the dominant portion of halloysite (H5L1) caused a systematic increase in the value of the E’ modulus.

The decrease in storage modulus in the temperature range from 30 °C to 70 °C was the smallest compared to PVC and the other composites with a halloysite and halloysite–lignin 5:1 wt/wt system, regardless of the filler concentration in the matrix; at 2.5 wt% concentration, the E’ value of PVC/H5L1 composites decreased by 17%, while that of PVC/H decreased by 19.4%. At the same filler content, the decrease in the modulus values of PVC/L and PVC/H1L5 samples was slightly greater than for PVC, for which a 20.3% decrease in E’ was recorded. It should be added that the decrease in the stiffness of composites with increasing temperature was greater the higher the filler content in the matrix, but in each case, the decrease in the E’ values of composites with 10 wt% filler content was smaller than that for PVC. This behavior allows us to claim that PVC/HL composites are better materials for the production of elements that should maintain stiffness.

Based on the analysis of the glass transition temperature values taken as values of the temperature in which the loss factor reveals the maximum value (Figure 8b) of the different composite materials, the fillers had no significant effect on changing the temperature range of the glass transition. The introduction of halloysite into the PVC matrix resulted in lower Tg values by approximately 1–2.1 °C, while in the case of PVC/L, these values were similar to those of PVC. The lower or unchanged glass transition temperature values compared to neat PVC were recorded for composites with hybrid fillers. Only in the case of composite samples containing 7.5 wt% halloysite–lignin fillers alone, and, additionally, in the PVC/10H1L1 sample, the T_g_ value was higher by approximately 1–2 °C compared to PVC. It should be added, however, that the differences in T_g_ values of all composites and unfilled PVC were no greater than 2.2%. Based on this, it can be concluded that halloysite–lignin hybrid filler does not significantly alter the mobility of PVC macromolecules.

**Figure 9:**
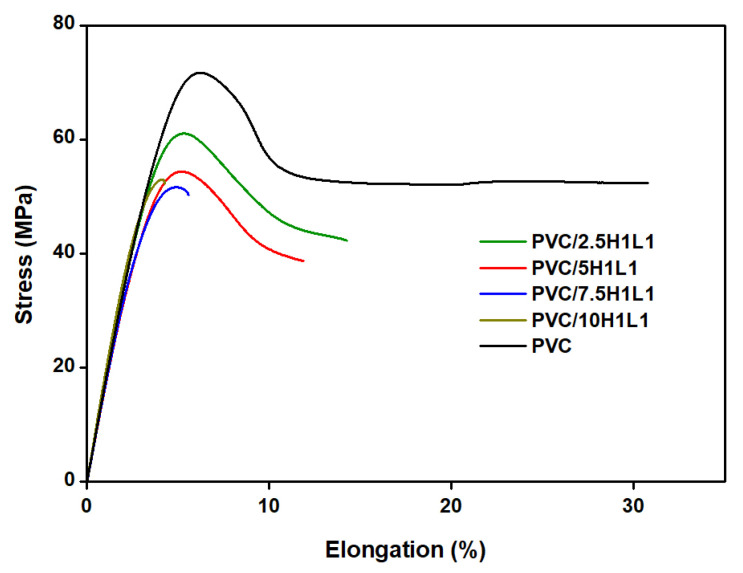
Representative stress–strain curves from the tensile test for the PVC and PVC composites with various concentrations of halloysite–lignin hybrid filler (1:1 wt/wt).

Figure 9 shows exemplary stress–strain curves for PVC and PVC composites with different loading of halloysite–lignin hybrid filler of equal content of both components. The mechanical properties determined based on the static tensile tests, i.e., Young’s modulus (E_t_), stress at yield point (σ_Y_), tensile strength (σ_M_) and strain at break (ε_B_) for all samples PVC and PVC composites, are summarized in Table 5.

In the case of neat PVC and PVC composites containing 2.5 wt% and 5 wt% of H1L1 filler, plastic deformation with a ductile-type fracture is present. There is a clear yield point on the curves, and the stress value at this point is the same as the tensile strength (δ_Y_ = δ_M_).

For PVC composites containing halloysite and lignin, over the entire range of concentrations used, as well as for hybrid fillers with component ratios of 1:5 and 5:1 introduced into the matrix at concentrations up to 7.5 wt%, similar stress–strain behavior is observed. An increase in the concentration of H1L5 and H5L1 fillers up to 10 wt% changes the fracture behavior to brittle-like; no yield point is observed on the curves. This fracture character appears in PVC/H1L1 composites with as low as 7.5 wt% filler content. It should be added that the tensile strength of PVC composites is lower than the δ_M_ value of unmodified PVC. PVC/H1L1 composites with 7.5 wt% and 10 wt% filler exhibit the lowest tensile strength, their values being 15 MPa lower than those of PVC.

A similar effect of a transformation from ductile to brittle-like fracture was found in our previous work [41] and was related to the introduction of a silica–lignin hybrid filler into the PVC matrix at concentrations equal to 5 wt% and higher.

The values of E_t_ show that the incorporation of halloysite and hybrid fillers, regardless of their composition and concentration in the matrix, leads to improvement in Young’s modulus compared to pristine PVC. The addition of 2.5 wt% and 5 wt% lignin causes insignificant deterioration in E_t_, but with a further increase in filler concentration, an increase in modulus is observed, the value of which for PVC/7.5L and PVC/10L composites is higher than that of pure PVC. The PVC/H1L5 composite samples have the highest modulus, with values in the range of 1800–1880 MPa and, therefore, 10–15% higher compared to PVC (1630 MPa). Thus, the fillers proposed in our study (in addition to lignin used in low concentrations) cause a slight stiffening of the PVC composites.

The effect of stiffness enhancement, already observed at a filler concentration of 2.5 wt%, especially in the case of PVC/H1L1 and PVC/H1L5, is accompanied by an almost two-fold increase in the value of elongation at break. Similar values of ɛ_B_ are exhibited by samples with the same filler content of H5L1, although the value of Young’s modulus, in this case, is practically the same as that of the PVC samples. A particularly high value of elongation at break can be found when stretching composites containing 2.5 wt% and 5 wt% halloysites; the value of ɛ_B_ in these cases is almost 8 times and 2.5 times higher than the elongation of pure PVC.

For all composite samples, elongation at break decreases with increasing filler concentration to 10 wt%, as reported in many works on polymer composites with natural and mineral fillers, including lignin-based hybrid fillers [41,43,82,83,84].

The high values of elongation at break found for composites with all types of fillers at the lowest concentration used are accompanied by high values of Charpy impact strength (see Table 5).

For composites with 2.5 wt% halloysite content, the impact strength is almost double compared to the a_cU_ value for neat PVC, which is 15.6 kJ/m^2^. Composites with the same content of hybrid fillers have impact strengths ranging from 19.5 kJ/m^2^ for samples with H5L1 filler to 23.4 kJ/m^2^ for samples with H1L1 filler; these values are approximately 4% to 25% higher compared to the impact strength of PVC/2.5L composites.

The increase in impact strength of the composite samples with a concomitant increase in elasticity found on the basis of a tensile test may indicate a toughness effect related to the introduction of a small amount of both halloysite–lignin hybrid fillers and single components into the matrix. At the same time, in the case of PVC/H1L1 and PVC/H1L5 composites, an increase of 10% in Young’s modulus values was found, recorded already at 2.5 wt% filler content, testifying to a slight increase in stiffness compared to unmodified PVC. The two effects (reinforcement-toughening) related to the introduction of 1 wt% of calcined halloysite into the PVC matrix were observed in our earlier work [81].

Similarly, as in the case of elongation at break, with the increasing content of all types of fillers, the impact strength decreased, reaching the lowest value for composites with 10 wt% of the filler, which was 6.1 kJ/m^2^ for PVC/10H and 5.9 kJ/m^2^ for PVC/10H1L1, respectively. For composites with lignin, the reduction in impact strength with increasing lignin content in the matrix was more gentle; the a_cU_ value for PVC/10L samples was 11.6 kJ/m^2^. It can be assumed that the increase in impact strength observed at low filler content was related to their uniform dispersion in the polymer matrix and good adhesion at the polymer–filler interface. Higher filler content can cause entanglement of lignin fibers and their partial agglomeration and, in the case of halloysite, agglomeration of particles, resulting in stress accumulation [45,47,85].

#### 3.2.4. Microscope Observations

**Figure 10:**
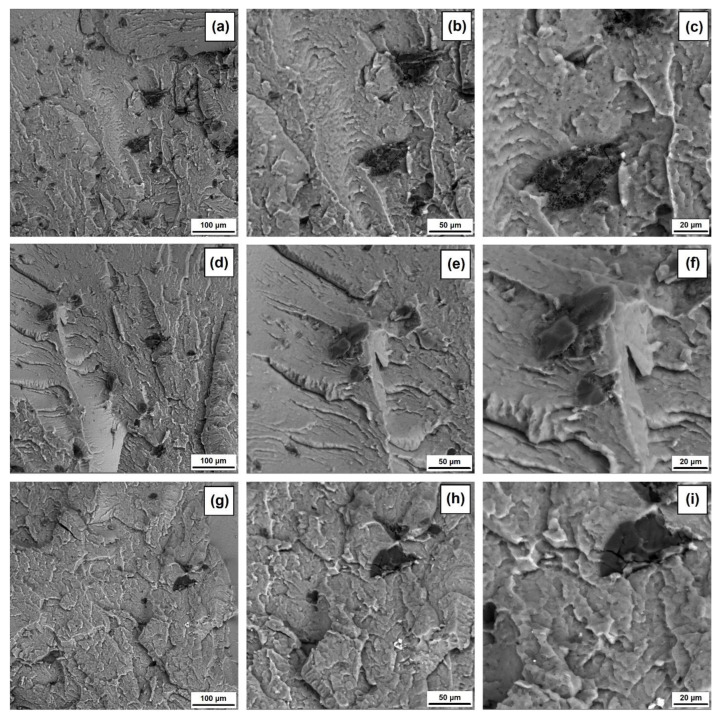
SEM images for PVC with 5 wt% of halloysite–lignin hybrid materials with the following component proportions: 1:5 wt/wt (**a**–**c**), 1:1 wt/wt (**d**–**f**) and 5:1 wt/wt (**g**–**i**).

**Figure 11:**
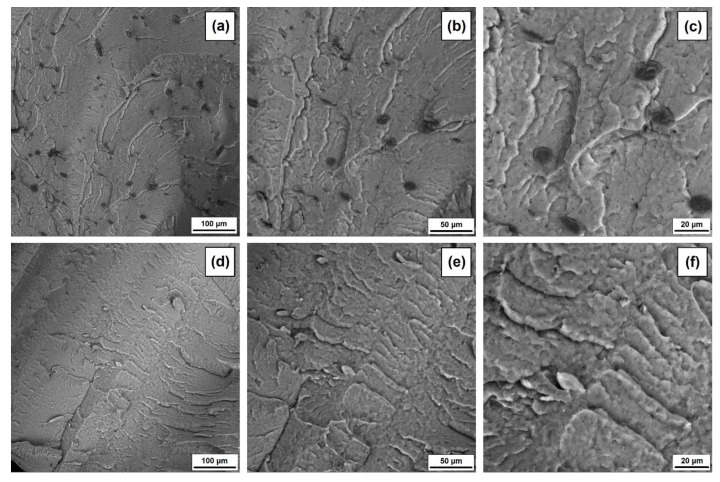
SEM images for PVC with 5 wt% of lignin (**a**–**c**) and halloysite (**d**–**f**) at different magnifications.

The SEM images of PVC composites with halloysite–lignin hybrid fillers with different ratios of the two components and with lignin and halloysite separately (see Figure 10 and Figure 11) show a layer structure of fracture surface, characteristic of gelated PVC. No inclusion of PVC-grain elements is observed, indicating a complete fusion of the polymer matrix in correctly selected conditions of processing.

The individual particles of the halloysite–lignin hybrid filler are visible in the SEM micrographs, and their number and size depend on the proportion of the two components of the hybrid filler. On the fracture surface of the PVC sample containing filler with the proportion of halloysite–lignin 1:5 wt/wt (Figure 10a–c), more dark-colored particles of varying sizes of approximately 10 µm to 50 µm are visible. These are, probably, agglomerates of individual smaller particles and are characterized by uneven shapes and surfaces. It should be added that particles of smaller sizes are evenly distributed on the surface of the fracture. Additionally, light-colored particles are visible with a size of approximately 1–2 µm associated with the presence of halloysite, which occurs individually, or on the surface of lignin particles, which is clearly visible in Figure 10c. The lower the proportion of lignin in the hybrid filler, the lesser the number of dark-colored particles, but individual agglomerates with a size and shape similar to the case of the PVC/H1L5 sample are visible (see Figure 10d–i). It may be seen that these particles are stably “embedded” in the polymer matrix, and there is no clear polymer–filler interfacial boundary, as seen in the image of the PVC/L sample shown in Figure 11a–c. The spherical lignin particles with a size of 10–20 µm and a smooth surface are evenly distributed on the surface of the fracture. The images of the PVC/H sample (see Figure 11d–f) show individual halloysite agglomerates with a length of approximately 20 μm, around which voids are visible. Their presence may indicate insufficient adhesion at the polymer–halloysite phase boundary.

## 4. Conclusions

Functional halloysite–lignin hybrid materials were designed and produced. Pristine components were used for their production, i.e., halloysite and lignin, between which, as a result of the use of a mortar grinder and a planetary ball mill, weak physical interactions in the form of hydrogen bonds were generated. This is evidenced by slight shifts in the absorption maxima of individual bands, visible in the analysis of FTIR spectra. In this way, class I hybrid materials were created. Moreover, the obtained materials are characterized by reasonably good homogeneity, as evidenced by the analysis of SEM images and the results of particle size distributions. Additionally, the hybrids have relatively good thermal stability, especially in the initial temperature range. All used hybrid fillers also show good electrokinetic stability over a wide pH range.

Processing rigid PVC with halloysite–lignin hybrid systems with a content of up to 10 wt% by the kneading method allowed us to obtain composites with a relatively homogeneous structure confirmed by SEM observations. The evident influence of HL systems in the concentration of 2.5 wt% on the PVC gelation properties was found.

For all PVC/HL composites, a slight enhancement of Young’s modulus compared to neat PVC was found. Moreover, samples containing 2.5 wt% of HL filler were noted to be characterized by twice the elongation at break, which, in combination with the high impact strength value, may provide information regarding the toughness effect and signifies uniform dispersion of HL systems in the PVC matrix as well as good adhesion at the polymer–filler interface. Thus, these materials can be used as impact modifiers that do not cause a decrease in stiffness, unlike plasticizers or lubricants.

The hybrid systems with a halloysite to lignin ratio such as 5:1 appear to be optimal for use as PVC filler. The favorable processing, as well as thermal properties (especially VST) and also mechanical properties of the produced composites, are guaranteed already by 2.5 wt% content of this material while ensuring the possibility of safe processing without the risk of degradation.

The beneficial effect of lignin as a component of the hybrid filler in the system with halloysite on the properties of rigid PVC is the rationale for further research on the use of biomass for the production of hybrid fillers with a mineral component. The use of lignocellulosic waste biomass as a low-value byproduct from various industrial sectors combined with a mineral component derived from natural deposits might be an eco-efficient solution for the sector of new composite materials with favorable processing and performance properties.

## Figures and Tables

**Table 1:** Compositions of the PVC-based composites.

Sample Name	Filler Type	Filler Content, wt. %
PVC		
PVC/2.5H	halloysite	2.5
PVC/5H		5
PVC/7.5H		7.5
PVC/10H		10
PVC/2.5L	lignin	2.5
PVC/5L		5
PVC/7.5L		7.5
PVC/10L		10
PVC/2.5H1L5	halloysite–lignin (1:5 wt/wt)	2.5
PVC/5H1L5		5
PVC/7.5H1L5		7.5
PVC/10H1L5		10
PVC/2.5H1L1	halloysite–lignin (1:1 wt/wt)	2.5
PVC/5H1L1		5
PVC/7.5H1L1		7.5
PVC/10H1L1		10
PVC/2.5H5L1	halloysite–lignin (5:1 wt/wt)	2.5
PVC/5H5L1		5
PVC/7.5H5L1		7.5
PVC/10H5L1		10

**Table 2:** Particle size distributions and polydispersity indexes for halloysite–lignin hybrid materials and pristine components (lignin and halloysite).

Sample	Particle Size Distribution (nm)	Polydispersity Index
Halloysite–lignin (1:5 wt/wt)	142–459	0.218
Halloysite–lignin (1:1 wt/wt)	106–615	0.377
Halloysite–lignin (5:1 wt/wt)	91–459; 1281–1990	0.452
Lignin	164–342; 459–6439	0.988
Halloysite	68–396; 1281–3580	0.654

**Table 3:** TGA analysis results for PVC and PVC composites.

Sample Name	Temperature of Weight Loss (°C)				RM at 700 °C (%)
	T_1%_	T_5%_	T_10%_	T_50%_	
PVC	250.5	266.3	276.1	316.0	18.1
PVC/2.5H	250.1	269.3	280.5	324.5	21.3
PVC/5H	243.7	267.7	278.9	325.3	21.5
PVC/7.5H	240.3	266.7	278.2	325.9	24.0
PVC/10H	238.1	266.1	278.9	325.1	26.2
PVC/2.5L	256.1	273.6	283.1	325.5	18.3
PVC/5L	261.1	276.6	285.5	330.3	19.9
PVC/7.5L	259.7	281.3	291.6	336.3	22.1
PVC/10L	260.1	277.3	287.1	330.3	22.9
PVC/2.5H1L5	253.9	271.1	282.2	326.4	18.7
PVC/5H1L5	256.3	273.9	285.0	328.6	20.5
PVC/7.5H1L5	257.9	273.7	283.5	327.3	22.1
PVC/10H1L5	252.3	274.7	284.2	330.1	22.5
PVC/2.5H1L1	251.9	269.5	280.5	324.7	20.0
PVC/5H1L1	253.5	272.3	283.0	327.2	21.1
PVC/7.5H1L1	253.1	270.7	281.3	328.8	21.7
PVC/10H1L1	255.5	272.7	283.9	330.0	23.9
PVC/2.5H5L1	249.5	269.3	279.7	324.6	19.8
PVC/5H5L1	243.1	263.3	276.6	321.3	20.6
PVC/7.5H5L1	242.3	264.0	275.9	322.0	22.3
PVC/10H5L1	242.3	264.7	276	323.6	24.3

**Table 4:** The storage modulus (E’) and glass transition temperature (T_g_) of PVC and PVC composites.

Sample Name	E’ (MPa)			T_g_ (°C)
	30 °C	50 °C	70 °C	
PVC	3136	2979	2548	94.7
PVC/2.5H	3263	3120	2712	92.6
PVC/5H	3698	3525	3066	92.9
PVC/7.5H	3764	3616	3189	93.7
PVC/10H	4027	3828	3234	92.7
PVC/2.5L	3265	3063	2733	93.9
PVC/5L	3514	3341	2823	94.3
PVC/7.5L	3582	3389	2888	94.7
PVC/10L	3565	3379	2938	94.5
PVC/2.5H1L5	3191	3043	2613	93.9
PVC/5H1L5	3229	3056	2638	94.5
PVC/7.5H1L5	3709	3523	3086	96.7
PVC/10H1L5	3747	3548	3056	94.4
PVC/2.5H1L1	3213	3063	2676	94.0
PVC/5H1L1	3249	3085	2674	94.4
PVC/7.5H1L1	3702	3525	3044	95.9
PVC/10H1L1	3749	3546	3049	95.7
PVC/2.5H5L1	3209	3054	2729	94.1
PVC/5H5L1	3360	3202	2763	93.7
PVC/7.5H5L1	3463	3281	2749	95.7
PVC/10H5L1	3795	3605	3128	94.7

**Table 5:** Mechanical properties of PVC and PVC composites with halloysite, lignin and halloysite–lignin hybrid fillers.

Sample	a_cU_ (kJ/m^2^)	E_t_ (MPa)	σ_Y_ (MPa)	σ_M_ (MPa)	ɛ_B_ (%)
PVC	15.6 ± 1.6	1630 ± 15.2	65.6 ± 10.8	65.6 ± 10.5	22.3 ± 12
PVC/2.5H	30.0 ± 5.7	1690 ± 19.5	58.6 ± 0.7	58.6 ± 0.7	174.7 ± 40.1
PVC/5H	15.6 ± 3.0	1740 ± 12.7	58.8 ± 0.3	58.8 ± 0.3	53.3 ± 5.2
PVC/7.5H	8.2 ± 1.1	1800 ± 12.2	58.9 ± 0.6	58.9 ± 0.5	6.9 ± 1.4
PVC/10H	6.1 ± 1.1	1840 ± 13.4	57.7 ± 0.5	57.7 ± 0.5	6.8 ± 1.6
PVC/2.5L	18.7 ± 0.9	1620 ± 10.7	56.1 ± 0.4	56.1 ± 0.4	47.8 ± 31.0
PVC/5L	17.6 ± 1.0	1620 ± 23.3	51.2 ± 1.1	51.3 ± 1.1	8.8 ± 1.0
PVC/7.5L	16.1 ± 2.3	1690 ± 8.9	52.8 ± 0.4	52.8 ± 0.4	16.4 ± 7.1
PVC/10L	11.6 ± 1.8	1730 ± 11.2	53.6 ± 0.5	53.6 ± 0.5	8.1 ± 2.4
PVC/2.5H1L5	21.2 ± 3.5	1800 ± 26.1	60.7 ± 0.8	60.7 ± 0.8	44.6 ± 31.4
PVC/5H1L5	16.8 ± 2.2	1830 ± 8.4	57.3 ± 0.2	57.3 ± 0.2	11.0 ± 4.6
PVC/7.5H1L5	11.7 ± 1.6	1880 ± 23.6	56.3 ± 0.7	56.3 ± 0.7	10.4 ± 1.0
PVC/10H1L5	11.0 ± 1.8	1860 ± 40.5	-	52.0 ± 1.6	4.7 ± 0.9
PVC/2.5H1L1	23.4 ± 3.1	1800 ± 16.1	61.1 ± 0.6	61.1 ± 0.6	45 ± 7.9
PVC/5H1L1	14.1 ± 2.4	1680 ± 22.3	56.4 ± 4.1	56.4 ± 4.1	10.3 ± 4.3
PVC/7.5H1L1	9.5 ± 1.6	1700 ± 35.1	-	51.5 ± 0.9	5.9 ± 1.9
PVC/10H1L1	5.9 ± 0.7	1760 ± 10.6	-	50.5 ± 1.6	4.7 ± 0.8
PVC/2.5H5L1	19.5 ± 2.9	1640 ± 14.3	57.3 ± 0.7	57.3 ± 0.7	49.8 ± 34.1
PVC/5H5L1	15.1 ± 1.3	1710 ± 14.2	56.8 ± 0.7	56.8 ± 0.7	19.6 ± 6.3
PVC/7.5H5L1	14.3± 2.4	1770 ± 17.5	56.8 ± 0.5	56.8 ± 0.5	11.9 ± 3.5
PVC/10H5L1	9.3 ± 0.9	1800 ± 10.3	-	55.4 ± 0.9	5.2 ± 0.8

## Data Availability

Not applicable.
